# Extension of the Human Fibrinogen Database with Detailed Clinical Information—The αC-Connector Segment

**DOI:** 10.3390/ijms23010132

**Published:** 2021-12-23

**Authors:** Zofie Sovova, Klara Pecankova, Pavel Majek, Jiri Suttnar

**Affiliations:** Department of Biochemistry, Institute of Hematology and Blood Transfusion, U Nemocnice 1, 12800 Prague, Czech Republic; pecankova@uhkt.cz (K.P.); majek@uhkt.cz (P.M.); suttnar@uhkt.cz (J.S.)

**Keywords:** fibrinogen, αC-connector, Human Fibrinogen Database, mutations, afibrinogenemia, hypofibrinogenemia, dysfibrinogenemia, hypodysfibrinogenemia

## Abstract

Fibrinogen, an abundant plasma glycoprotein, is involved in the final stage of blood coagulation. Decreased fibrinogen levels, which may be caused by mutations, are manifested mainly in bleeding and thrombotic disorders. Clinically relevant mutations of fibrinogen are listed in the Human Fibrinogen Database. For the αC-connector (amino acids Aα240–410, nascent chain numbering), we have extended this database, with detailed descriptions of the clinical manifestations among members of reported families. This includes the specification of bleeding and thrombotic events and results of coagulation assays. Where available, the impact of a mutation on clotting and fibrinolysis is reported. The collected data show that the Human Fibrinogen Database reports considerably fewer missense and synonymous mutations than the general COSMIC and dbSNP databases. Homozygous nonsense or frameshift mutations in the αC-connector are responsible for most clinically relevant symptoms, while heterozygous mutations are often asymptomatic. Symptomatic subjects suffer from bleeding and, less frequently, from thrombotic events. Miscarriages within the first trimester and prolonged wound healing were reported in a few subjects. All mutations inducing thrombotic phenotypes are located at the identical positions within the consensus sequence of the tandem repeats.

## 1. Introduction

### 1.1. Fibrinogen Structure

**Figure 1:**
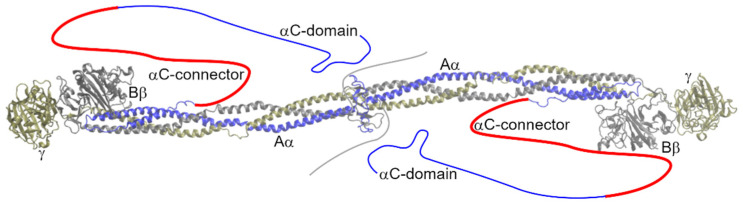
Structure of fibrinogen (3GHG; [5]). Missing parts of the molecule are sketched. The αC-connector is highlighted in red.

Fibrinogen (Figure 1) is a plasma glycoprotein that, in its major form, contains 2964 amino acids (AAs) and has a molecular weight of approximately 340 kDa. It is involved in the final stage of blood coagulation and participates, inter alia, in inflammation, cell migration, and tumorigenesis [1,2,3,4].

Fibrinogen consists of two copies of the chains Aα (629 AAs; numbering and count according to the nascent chain), Bβ (491 AAs), and γ (437 AAs). The chains are encoded by three genes, *FGA*, *FGB*, and *FGG*, all of which are located in an approximately 50 kb region on chromosome 4 (4q31.3–4q32.1) [6]. Fibrinogen is predominantly synthesized in hepatic parenchymal cells, with the Bβ chain considered the rate-limiting chain [7]. During assembly these chains form heterotrimers and two such trimers consecutively interlink in a centrosymmetric manner with their N-termini oriented towards each other [5]. Fibrinogen is co-translationally glycosylated before entering the secretory pathway [8]. Seventeen interchain disulfide bridges link its chains together, and 12other intrachain disulfide bridges maintain proper tertiary structure. The fibrin monomer is formed by the thrombin cleavage of fibrinopeptides A and B from the N-termini of the fibrinogen Aα and Bβ chains. Unlike fibrinogen, the fibrin monomer readily polymerizes and forms a network, called a fibrin clot, that is among the major components of blood clots and thrombi.

The disordered N-termini of all chains are followed by a triple, parallel coiled-coil domain. The Bβ and γ chains further form a globular fibrinogen-related domain; its structure is described, e.g., in works [9,10]. The Aα chain loops back, making a short α-helix of 17 AAs that is anti-parallel to the coiled-coil domain with which it interacts in a hydrophobic manner. The last part of the Aα chain is known as the αC-region (Aα240–629, nascent chain numbering). This region is divided into the N-terminal αC-connector (Aα240–410) and C-terminal αC-domain (Aα411–629) [11]. The αC-domain interacts with the N-termini of fibrinogen but not of fibrin [12]. Intermolecular interactions among unbounded αC-domains enhance the lateral aggregation of fibrin protofibers. The β-hairpin is the dominant structural feature of the αC-domain [13]. The αC-connector, the subject of this review, is characterized by 10 tandem repeats with a period of 13 AAs at its C-terminus. The copy number and period of tandem repeats vary among species. The αC-connector contributes to the flexibility of fibrin fibers [14]. Crystal structures did not reveal any secondary structures in this region (secondary structures were absent in fibrinogen crystal structures, suggesting that it does not have any extensive secondary structure), although measurements of the heat capacity functions and circular dichroism spectra suggest that the αC-connector adopts the extended helical poly (L-proline) type IIconformation (PPII) [15].

### 1.2. Congenital Fibrinogen Diseases

Like all proteins, fibrinogen is subject to mutagenesis. If a mutation significantly affects fibrinogen structure, the protein is not released into the blood. The state of having an undetectable amount of fibrinogen in the blood is called afibrinogenemia and usually originates from homozygous nonsense or frameshift mutations. Hypofibrinogenemia is diagnosed when the detectable level of plasma fibrinogen is below physiological levels. Fibrinogen with little structural impairment can overcome control mechanisms and be released into the blood even though it is not fully functional. Such a condition is called dysfibrinogenemia. Hypodysfibrinogenemia refers to a state in which the levels of fibrinogen are decreased and at least some molecules are impaired. Hypofibrinogenemia and hypodysfibrinogenemia are further divided into the severe (level of functional resp. antigenic fibrinogen below 0.5 g/L), moderate (0.5 to 0.9 g/L), and mild (1 g/L to lower limit of normal value) types [16]. These four diseases are collectively referred to as congenital fibrinogen disorders (CFDs).

CFDs are characterized by bleeding or, less frequently, by thrombotic episodes, or they can be asymptomatic. Afibrinogenemia is characterized by heavy bleeding episodes. Intracranial bleeding is the most frequent cause of death in afibrinogenemic patients [17]. Some women suffer from prolonged, heavy menstrual bleeding, pre- and post-partum bleeding, miscarriages within the first trimester of gestation, and placental abruption. Ischemic stroke and deep venous thrombosis (DVT) are the most common thrombotic episodes in afibrinogenemic patients. Venous or arterial thromboses appear less frequently. Bone cysts, spleen rupture, and impaired wound healing are occasionally reported [18,19,20].

Hypofibrinogenemia is usually asymptomatic, especially in patients with fibrinogen levels higher than 1 g/L. Spontaneous bleeding episodes become more frequent with decreasing levels of fibrinogen. In women, menorrhagia is frequent but miscarriages are not reported. Mutations in certain parts of the fibrinogen-related domain may result in fibrinogen storage disease [21].

Dysfibrinogenemia may manifest as mild bleeding tendencies and venous and arterial thrombosis. Women may experience first trimester miscarriages, placental abruption, or post-partum thrombosis. Dysfibrinogenemia may cause inherited thrombophilia or severe complications of pulmonary embolism. However, the majority of dysfibrinogenemic patients are asymptomatic [22,23].

Hypodysfibrinogenemia combines the manifestations reported for dysfibrinogenemia and hypofibrinogenemia. Patients are frequently asymptomatic but may also suffer from thrombotic and bleeding symptoms. Spontaneous bleeding occurs in subjects who have levels of functional fibrinogen below 0.7 g/L. Women report heavy menstrual bleeding [24].

Some mutations of the Aα536–574 region cause renal amyloidosis. This rare disease is characterized by fibrinogen accumulation in the kidneys and does not belong among CFDs as it does not influence the levels of plasma fibrinogen [25].

### 1.3. Mutation Databases

Mutations associated with CFDs are collected in the Human Fibrinogen Database (HFD) [26]. This database lists mutation positions at the protein and nucleotide level (coding and genomic coordinates are mixed), its zygosity, diagnosed CFD, whether the mutation is associated with bleeding or thrombosis, and the reference to the original report. All data were accepted from the referred works, and they were not further curated. Thus, the reports may be incomplete or even inaccurate in some cases, and the reference to the original work in the form “first_author’s_surname, year_of_publication” is not always explicit (e.g., the reference “Asselta, 2015” may point to her works published in both Thrombosis Research [27] and Thrombosis and Haemostasis [28]). Additionally, detailed information about the clinical manifestations (e.g., location of the bleeding site or type of thrombotic event) and severity of the CFD, confirmed by the results of laboratory examination, is missing.

The COSMIC database was originally intended to collect cancer-associated mutations [29] but recently was converted to a general database [30]; dbSNP [31] is another general database. Both these databases collect information about the position and abundance of mutations, although clinically and structurally relevant information is missing.

This work is the first step in an ongoing effort to extend the HFD with detailed information about the clinical manifestations of each mutation, including the severity of diagnosed CFD. It focuses on mutations in the αC-connector of the Aα chain of fibrinogen. If available, information about the influence of a mutation on fibrin polymerization and fibrin clot architecture is included. The position of each mutation is defined at the protein (both mature and nascent chain), coding, and genomic level. We supplied the missing items and adjusted the reported positions to be complementary at all levels if needed. We also reported relevant information not only about the patient but also about all reported participants of the clinical study, usually the patient’s relatives.

## 2. Mutations in the αC-Connector of Fibrinogen

The COSMIC, dbSNP, and HF databases include 404 unique nucleotide mutations in the αC-connector of fibrinogen (c.718–1230; g.3993–4505; see Table S1). This region also contains a complex compound mutation, known as fibrinogen Champagne au Mont d’Or [32], that is considered one mutation for the purposes of this review. This trans compound comprises 12 independent mutations in a region of 200 bp, including a 117 bp duplication (see Figure S1). Sixteen of the 404 mutations are deletions (including two in-frame deletions), nine are insertions (including two in-frame insertions and two duplications), one (c.991A>G) is reported as a single nucleotide polymorphism (SNP; a mutation occurring at least in 5% of the population), and the others are single point substitutions.

**Figure 2:**
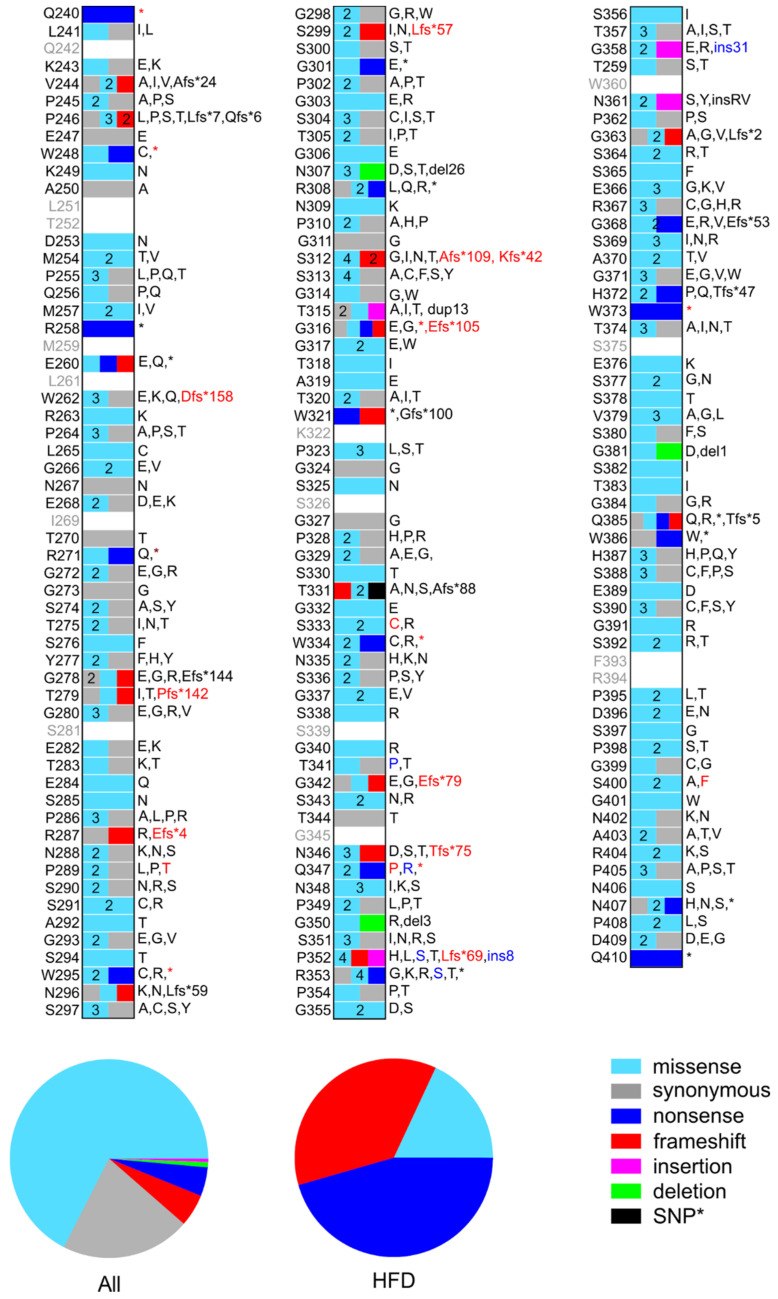
Schematic representation of the position and nature of mutations in the αC-connector of fibrinogen reported in the dbSNP, COSMIC, and HF databases. Mutations reported in the HFD are indicated by red labels. Mutations belonging to the fibrinogen Champagne au Mont d’Or are indicated by blue labels, although some of them are reported as independent mutations in dbSNP. The plots below compare the percentage of each type of mutation in all databases and the HFD only. *SNP AαT331A is not considered in these plots.

At the protein level, these 404 nucleotide mutations are translated into 380 unique mutations (see Figure 2 and Table S1). Eighty of these are synonymous mutations, 257 are missense mutations including one SNP, 20 are frameshift mutations, 18 are nonsense mutations, three are in-frame deletions, and two are in-frame insertions. Twelve of these mutations are reported as compound mutations. Fibrinogen Champagne au Mont d’Or, per se, is translated into five missense mutations and two in-frame insertions of eight and 31 AAs, respectively.

We are aware that 8.3% resp. 17% of especially older records in the dbSNP can be false positives [33,34]. This means that up to 69 unknown records in the database may be redundant. From a statistical point of view, listing these up to 69 possibly erroneous mutations will cause a smaller error than neglecting the 335 true-positive mutations. For these reasons, we consider the mutations reported in COSMIC and dbSNP.

Both dbSNP and COSMIC gather mutations from various sources, including whole-genome sequencing projects working with healthy subjects. Although some entries are incorporated from clinical databases, like the HFD, numerous mutations in the αC-connector must be asymptomatic or have very mild clinical manifestations that go unnoticed. A possible explanation is that these mutations are heterozygous, as heterozygous mutations in the αC-connector are frequently asymptomatic. However, these mutations may also be homozygous and have no effect on coagulation. It is impossible to distinguish between these two possibilities because dbSNP and COSMIC do not report the zygosity and clinical manifestations of the mutations therein.

## 3. Detailed Characterization of Mutations in the αC-Connector

This section deals with the mutations in the αC-connector reported in the HFD because they are usually broadly characterized in the original literature. These mutations are summarized in Table 1 and Table S2 and Figure 2 and Figure S2. The AαT331A mutation is discussed in a separate section since it is the only known SNP in the αC-connector of fibrinogen.

When preparing Table 1 and Table S2, we found discrepancies in the mutation locations at the protein and nucleotide levels. Consequently, we cross-checked the protein (reference NP_068657.1), c.DNA (coding region of NM_021871.4 that starts with the 56th nucleotide of the reference sequence), and genomic (M64982.1) coordinates of all mutations in the αC-connector reported in the HFD and in the respective papers. We completed missing entries and, if necessary, adjusted the values of the existing entries. Text S1 explains the adjustment done. Because the HFD was last updated on 1 June 2020, we searched for newer works dealing with mutations in the αC-connector using Google Scholar, Web of Knowledge, and PubMed [35]. The search was performed in July 2021 and no new reports were found. The mutation designation follows the recommendations of Den Dunnen et al. [36]. We use nascent chain numbering, including the 19 AA signal peptide for protein coordinates, in this work.

### 3.1. Characterization of Reported Subjects

Works dealing with mutations in the αC-connector mention 132 members of 35 families. There are 60 males and 48 females in the cohort. Sex is not reported for 24 subjects. Age at the time of participation in the referred study is available for 57 subjects and ranges from newborns (we defined their age and that of infants as 0 years for further statistics) to 83 years. The average age is 27.2 years, median is 27.0 years, and mode is 0 (eight subjects), i.e., they are either newborns or infants. Consanguinity is reported in 12 of these families and is explicitly denied in six families; the report is missing for 17 families.

Subjects come from 18 countries worldwide, namely the United States (19 members of three families), Turkey (18/3), Tunisia (15/3), Iran (13/3), Czech Republic (11/2), Egypt (8/1), France (8/4), Italy (7/4), Netherlands (7/1), China (6/1), India (5/3), Morocco (4/1), Serbia (3/1), South Korea (3/1), Germany (2/1), Bulgaria, Algeria, Switzerland, and Lebanon (all 1/1). These data demonstrate that CFDs are reported either from countries where consanguineous marriages are common or from countries with advanced and widely available medical care that can recognize CFDs with mild manifestations. 

### 3.2. Characterization of Mutations in the αC-Connector Reported in Studies

In the αC-connector, there are 22 unique nucleotide mutations and a complex trans compound mutation called fibrinogen Champagne au Mont d’Or. Twelve other mutations are also reported as compound mutations.

Mutations within the αC-connector are translated into 22 unique protein mutations as AαW334* is encoded in two ways (c.1001G>A or c.1002G>A). There are 10 frameshift mutations, eight nonsense mutations, and four missense mutations in the αC-connector.

In the HFD, a database focused on the impact of fibrinogen mutations on coagulation pathophysiology, there is a considerably higher proportion of nonsense and frameshift mutations than in the general dbSNP. This can be interpreted to mean that missense mutations do not induce sufficient changes in fibrinogen structure to restrain its secretion into the blood.

Mutations within the αC-connector (or at least one mutation of the compound mutation) are reported for 92 subjects, wild-type fibrinogen is reported for eight subjects, and information about the nature of the mutation is absent for 25 subjects, i.e., the family members of the patients. Seven other subjects have a sole mutation outside of the αC-connector. Thirty-two of the mutations in the αC-connector are homozygous, two are compound mutations, and 60 are heterozygous mutations including 10 compound mutations. While nonsense and frameshift mutations are reported as both homozygous and heterozygous, there is no report of homozygous missense mutations in the αC-connector.

### 3.3. Effect of Mutations in the αC-Connector at the Fibrinogen Level

The level of plasma fibrinogen was examined for 92 subjects. Afibrinogenemia was reported for 36 of them, dysfibrinogenemia for 13, hypodysfibrinogenemia for 7 and hypofibrinogenemia for 4 subjects. No CFD was reported for 31 subjects.

Mutations within the αC-connector were reported in 80 genetically tested subjects. There was at least one member of each family in this sub-cohort. We excluded the carrier of p.[G32E];[S333F] and a seven-member family carrying p.[G36C;S400F] as the mutation outside of the αC-connector is considered to cause dysfibrinogenemia [52,59], (see Section 3.5). Among this sub-cohort, there are 32 carriers of homozygous mutations (two compound mutations) and 39 carriers of heterozygous mutations (including eight compound mutations). Afibrinogenemia is diagnosed in 34 subjects carrying 15 unique mutations, dysfibrinogenemia in three subjects (all unique), hypofibrinogenemia in four subjects (three unique mutations), hypodysfibrinogenemia in seven subjects (four unique mutations), and no CFD in 24 subjects (nine unique mutations; see Table 2).

The zygosity of mutations influences their clinical manifestations considerably. Homozygous nonsense and frameshift mutations usually result in afibrinogenemia or severe hypofibrinogenemia. Carriers of heterozygous mutations usually have normal levels of fibrinogen. This confirms the in vitro-derived finding [7], that the Aα-chain is not the rate-limiting factor for fibrinogen assembly and secretion.

Afibrinogenemia is reported for carriers of homozygous nonsense or frameshift mutations and for subjects carrying cis compound heterozygous mutations where the nonsense or frameshift mutation in the αC-connector is accompanied by another mutation that restrains fibrinogen secretion into the blood (see Section 3.5). 

Dysfibrinogenemia is reported for heterozygous carriers of missense mutations and for subjects carrying fibrinogen Champagne au Mont d’Or, which contains heterozygous missense mutations together with in-frame insertions. Dysfibrinogenemia in an AαP347Q carrier is hypothesized to result from tissue plasminogen activator treatment. The hypothesis could not be verified due to the subject’s death. As two of the subject’s relatives carrying the same heterozygous mutations had normal levels of fibrinogen, the link between AαP347Q and congenital dysfibrinogenemia is questionable. Heterozygous carriers of the Champagne au Mont d’Or compound mutation and AαP289T are diagnosed with dysfibrinogenemia due to the presence of abnormal fibrinogen in the blood, although the values of functional and antigenic fibrinogen in these subjects are within the normal range. Note that no homozygous missense mutation is reported in the cohort; thus, it is impossible to draw any conclusions about such mutations.

Normal levels of fibrinogen are reported among heterozygous carriers of nonsense and frameshift mutations and among heterozygous carriers of AαP347Q.

Hypofibrinogenemia is rarely associated with mutations in the αC-connector, and there is no case of moderate hypofibrinogenemia reported in this region. Severe hypofibrinogenemia is reported for an AαR287Efs*4 carrier whose level of functional fibrinogen (0.06 g/L) is detectable but negligible. Mild hypofibrinogenemia is recognized in two carriers of the AαS312Afs*109 mutation (functional fibrinogen levels 1.64 g/L and 1.75 g/L; normal values from 1.9 g/L) and in a carrier of the p.[W334*;T331A];[T331A] mutation (functional fibrinogen 2.07 g/L; normal values from 2.5 g/L). For all three subjects, the level of fibrinogen approaches the normal value. Their relatives, who carry the same mutation (in the latter case accompanied by the heterozygous BβR478K SNP), exhibit normal levels of fibrinogen.

Hypodysfibrinogenemia, in its severe form, is reported for a homozygous AαW295* carrier (functional fibrinogen 0.2 g/L), three carriers of the heterozygous trans compound mutation p.[G342Efs*79;T331A] (functional fibrinogen between 0.2 and 0.28 g/L as measured by repeated tests), and a [p.Q347*;c.510+1G>T] carrier (functional fibrinogen 0.003 g/L for both patients). Two contradictory values for functional fibrinogen levels, 0.7 g/L by the Clauss method and 0 by the prothrombin time-derived method, are reported for a carrier of the homozygous AαW373* mutation. As the presence of the mutated fibrinogen in plasma was confirmed by western blot, we accepted the value obtained by the Clauss method and report this hypodysfibrinogenemia as moderate.

Every unique mutation of the given zygosity is associated with defined CFD except for two cases. Heterozygous AαS312Afs*109 is reported either in subjects with a normal level of fibrinogen or subjects with mild hypofibrinogenemia. Regardless of the fibrinogen level, heterozygous carriers of AαS312Afs*109 are asymptomatic. Afibrinogenemia or severe hypodysfibrinogenemia are reported for homozygous carriers of AαW295*.

While interpreting the clinical diagnoses associated with decreased levels of functional and/or total fibrinogen, one must be aware that these diagnoses are only discrete, manmade categories for a continuous scale of levels of fibrinogen in the blood. In other words, the state of a hypofibrinogenemic patient with a fibrinogen level of 1.45 g/L will be closer to that of a healthy individual with a fibrinogen level of 1.55 g/L (considering normal fibrinogen levels to be 1.5–4.0 g/L) than to that of another hypofibrinogenemic patient with a fibrinogen level of 0.25 g/L. It is also worth noting that normal levels of fibrinogen vary among laboratories. Table S3 helps the reader with a comprehensive overview of the severity of individual CFDs. One must also be aware that the limit of detection of various devices and techniques differs. Thus, a condition that is designated as afibrinogenemia (no detectable fibrinogen) by one lab may be considered severe hypofibrinogenemia (very low levels of fibrinogen) by another. To be able to diagnose dysfibrinogenemia and hypodysfibrinogenemia, the levels of both total and functional fibrinogen must be determined. This has not always been done, especially by the older studies. Thus, there is not enough information to distinguish normal fibrinogen levels from dysfibrinogenemia, and the reported hypofibrinogenemia may actually be hypodysfibrinogenemia.

### 3.4. Clinical Manifestations of Mutations in the αC-Connector

Information about the clinical manifestations of mutations within the αC-connector is available for 83 members of 25 families (Table 3 and Table S2). Reports contain medical information for subjects whose fibrinogen was not genetically tested. We treat these symptomatic family members as if they have the same mutations as their relatives, although we are aware that this does not always hold true.

Bleeding is the most common clinical manifestation of CFDs caused by mutations in the αC-connector of fibrinogen. It was reported for 43 subjects. Thrombotic complications were reported for 11 subjects. No subjects report bleeding tendencies together with thromboses unless they received fibrinogen prophylaxis before the thrombotic event occurred. Women may suffer from (repetitive) miscarriages (five subjects) and two subjects reported delayed wound healing. Another 32 subjects were asymptomatic.

#### 3.4.1. Bleeding Phenotype

Bleeding was reported for 43 subjects regardless of their diagnosis and the mutation type. Umbilical cord bleeding was reported for 15 out of 36 afibrinogenemic subjects and for two hypodysfibrinogenemic siblings who have trace amounts of functional fibrinogen in the blood. Other frequently reported types of spontaneous bleeding are epistaxis (10 subjects), bleeding from the mouth including gums (six subjects carrying either the AαQ240* or AαS312Afs*109 homozygous mutations), and bruising (five subjects). Subjects further report hemarthrosis, muscle bleeding, skin bleeding, mucous membrane bleeding, intracranial bleeding, petechiae, hematemesis, and hematuria. Spontaneous bleeding was reported or suspected to be the cause of death in seven subjects who were not examined in connection with CFDs. Induced bleeding (postoperative or posttraumatic) was reported for 22 subjects, 11 of whom also reported spontaneous bleeding.

#### 3.4.2. Thrombotic Phenotype

The thrombotic phenotype was reported for 11 carriers of five unique mutations. Two of them reported thrombotic events after fibrinogen prophylaxis.

The carrier of the fibrinogen Champagne au Mont d’Or (i.e., the complex compound mutation) reported DVT and pulmonary embolism. There were certain indications for thrombotic complications in his mother, who reported prolonged post-partum phenindione treatment.

DVT and myocardial infarction were reported in two unrelated afibrinogenemic carriers of homozygous AαW295*. Another carrier of this mutation reports bleeding. This subject was diagnosed with severe hypodysfibrinogenemia.

Thrombotic tendencies were reported in a consanguineous family exhibiting the p.[W334*;T331A] allele. Its homozygous carrier reported spontaneous DVT. Independent thrombotic complications appeared during long-lasting treatment for the narrowing of arteries in his arm. Three of his father’s siblings, who were not genetically tested, died of ischemic stroke. Thrombosis and thrombophlebitis were reported in a homozygous carrier of the AαW334* mutation from another family. This subject was on fibrinogen prophylaxis to prevent bleeding (and possible miscarriage) during pregnancy. Note that the AαW334* mutation was encoded by different nucleotides in these two families (c.1001G>A and c.1002G>A). There is not enough data to examine the potential impact of the genomic position of the mutation on its pathophysiological manifestation.

Myocardial infarction was reported as the cause of death for a subject carrying the heterozygous AαQ347P mutation. His two relatives, carriers of the same mutation, were asymptomatic.

Thrombosis and pulmonary embolism after fibrinogen prophylaxis were reported for a [p.Q347*];[c.510+1G>T] carrier, who otherwise reported posttraumatic bleeding. His sister, a carrier of the same compound mutation, reported posttraumatic bleeding. Two other members of this family, carrying heterozygous AαQ347*, were asymptomatic.

Interestingly, all mutations associated with the thrombotic phenotype were located in the tandem repeat-containing part of the αC-connector. Not considering the complex fibrinogen Champagne au Mont d’Or, all the mutated AAs (AαW295, AαW334, and AαQ347) were situated at the respective positions within the consensus sequence of the tandem repeats. It is questionable whether this is a coincidence or whether the last position in the consensus sequence of the tandem repeat is of importance for fibrinogen behavior.

#### 3.4.3. Women’s Health

In the cohort, there were 21 women old enough to have menstrual bleeding or become pregnant (16 years and older). Five of them reported at least one miscarriage. If reported, the miscarriages occurred within the first trimester of pregnancy. Four of these women suffered from afibrinogenemia or a severe type of hypofibrinogenemia or hypodysfibrinogenemia. The fifth woman, a heterozygous AαW334* carrier, had normal levels of fibrinogen. Apart from a miscarriage, she also reported bleeding.

Pre- or post-partum bleeding was reported by three women (two homozygous afibrinogenemic AαW334* carriers and a dysfibrinogenemic carrier of compound heterozygous [p.G32E];[p.S333F]). Five women reported menorrhagia. They included two afibrinogenemic carriers of homozygous AαQ240*, a heterozygous carrier of AαW334* with a normal level of fibrinogen, and a p.[G32E];[S333F] carrier. In a p.[G36C;S400F];[T331A] carrier, menorrhagia was the only manifestation of dysfibrinogenemia.

Note that an AαW334* heterozygous carrier, who had normal levels of functional fibrinogen (2.4 g/L), reported epistaxis, menorrhagia, and a miscarriage. Three other heterozygous family members, who had normal levels of fibrinogen, were asymptomatic.

#### 3.4.4. Delayed Wound Healing

Delayed wound healing was reported for two afibrinogenemic subjects. Both of them were homozygous carriers of the AαQ240* or AαW334* nonsense mutations, and bleeding was the major manifestation of their disease.

#### 3.4.5. Asymptomatic Subjects

The 30 remaining subjects were asymptomatic. None of them carried a homozygous mutation or were diagnosed with afibrinogenemia and hypodysfibrinogenemia. Most of them (17) did not suffer from any CFD. Fibrinogen level was not reported for seven subjects. One dysfibrinogenemic subject, a heterozygous AαP289T carrier, had normal levels of fibrinogen. Dysfibrinogenemia was reported for three asymptomatic p.[G36C;S400F] carriers. Two asymptomatic carriers of heterozygous AαS312Afs*109 suffered from mild hypofibrinogenemia, although other carriers of this mutation had normal fibrinogen levels.

### 3.5. Compound Mutations with at Least One Mutation Located in the αC-Connector

Twelve out of 23 unique protein mutations reported in the HFD (fibrinogen Champagne au Mont d’Or is treated as a single mutation) were expressed as a compound mutation at least in one subject. Considering the manifestations of these mutations, the influence of each mutation must be taken into account. To deal with this issue, we briefly reviewed the effects of the other mutations that were reported together with the mutations within the αC-connector (Table S4). We used the HFD as the only source for this sub-review and focused on the following mutations: Aα144Sfs*16, AαG32E, AαG36C, BβS189T, and intronic mutations c.510+1G>T (rs146387238), the “11-kb deletion” (RCV000017876.28), and c.510+36T>C. We do not deal with compound mutations containing the AαT331A SNP, i.e., mutations containing the alleles p.[W334*;T331A] and p.[G342Efs*79; T331A].

The mutation AαW334* was reported in a subject (A12 in [51]) together with AαK144fs*16 in a cis compound heterozygous form. The subject was diagnosed with afibrinogenemia (fibrinogen levels are not available). Both mutations shorten the C-terminus of the Aα-chain of fibrinogen. In the homozygous state, they caused afibrinogenemia that manifested as umbilical cord bleeding in the AαK144fs*16 carrier. In the heterozygous state, they do not influence fibrinogen levels [53,60]. In the reported p.[AαW334*];[AαK144fs*16] carrier, both alleles produce shortened Aα-chains that were not secreted into the blood. This condition is diagnosed as afibrinogenemia.

The same explanation is used for a cis compound heterozygous carrier of AαS312Afs*109 together with an “11-kb deletion” (B6 in [61,62]) and for all four cis compound heterozygous mutations containing c.510+1G>T [19,63,64,65,66,67] with AαT279Pfs*142, AαG316*, AαP352Lfs*69, or AαQ347*. These mutations are diagnosed as afibrinogenemia, except for AαQ347*; c.510+1G>T, which is reported as severe hypodysfibrinogenemia with trace amounts of both functional and antigenic fibrinogen. In the homozygous state, both c.510+1G>T and the “11-kb deletion” manifest as afibrinogenemia; thus, there is no Aα-chain synthesized by the other allele that could compensate for the lack of fibrinogen resulting from the abovementioned nonsense and frameshift mutations. Both intronic mutations in homozygous form are associated with a bleeding phenotype. A carrier of c.510+1G>T reported bone cysts.

In addition to the homozygous AαR271* mutation, a heterozygous BβS189T mutation was also present in a patient with afibrinogenemia [42]. The effect of the BβS189T mutation that is not reported by any other work is not discussed as the homozygous AαR271* mutation is sufficient for the induction of afibrinogenemia.

The AαS333C mutation is reported as a cis compound heterozygous mutation with AαG32E [52]. The subject with these mutations suffered from dysfibrinogenemia that manifested as a bleeding tendency. The turbidimetric curve has a longer lag phase and smaller maximum turbidity than the control, and fibrinolysis by thrombin is prolonged. The fibrin clot formed by the mutated fibrin exhibited wider fibers. There are no reports about AαS334C as a sole mutation, although we found two notes about the heterozygous AαG32E mutation [68,69] resulting in dysfibrinogenemia. The turbidimetric curve measured on fibrinogen containing the heterozygous AαG32E mutation has a longer lag phase and lower maximal turbidity. Data reporting the influence of the mutation on fibrin clots are ambiguous (no effect vs. clot with narrower fibers and bigger pores), as are the reports on the speed of fibrinolysis (no effect vs. faster lysis). Both works report a bleeding phenotype. Combined with the report of the compound heterozygous mutation, we find that AαS333C may be responsible for wider fibrin fibers, resulting in the prolongation of clot lysis.

The trans compound mutation p.[G36C;S400F] is reported to cause dysfibrinogenemia. Some family members also had the AαT331A SNP on their second allele. The compound mutation manifested as delayed fibrin polymerization and a decreased release of fibrinopeptide A. Dysfibrinogenemia was reported in family members without the SNP. This suggests that the contribution of AαS400F to the resultant phenotype, if any, is marginal. We did not find any other reports of the AαG36C mutation. As both pathophysiologies reported in the family were associated with hampered fibrinopeptide release, we agreed with the authors of the original work that there is no conclusive information about the effect of AαS400F on fibrinogen behavior.

The complex compound mutation Champagne au Mont d’Or [32] is reported as p.[T341P; Q347R; P352S; P352_353insGTGGTATW; R353K; T357S; G358_T359insPGSAGSWNSGSSGPGSTGNRNPGSSGTGGTA] and is associated with the thrombotic phenotype. Patient also carried the SNP AαT331A in a trans position and an intronic mutation, c.510+36T>C. We do not discuss this mutation further because information about individual mutations, but for the SNP AαT331A, is not available.

### 3.6. Impact of Mutations in the αC-Connector on Fibrin Clot Characteristics

Laboratory examinations of subjects’ blood usually report coagulation tests only (see Table S3). These values are reported to support the diagnosis of a subject with decreased fibrinogen levels. Five studies dealing with the mutations p.R287Efs*4, p.[G32E];[S333C], p.[G342Efs*79;T331A], [p.Q347*];[c.510+1G>T], and p.Q347P characterized fibrin polymerization as determined by absorbance measurements (Table 4). All studies reported a prolonged lag phase as well as a decreased maximum rate of polymerization (V_max_) and final turbidity, regardless of the diagnosis (ranging from severe hypofibrinogenemia to healthy) and zygosity of the mutation. Clottability is not affected by the homozygous p.Q347P mutation, in contrast to the p.[G342Efs*79;T331A] mutation, which decreases clottability by approximately 4.5-fold. However, three out of these four mutations are compound mutations, indicating that fibrinogen characteristics can be influenced by both mutations.

Changes in a subject’s fibrinogen characteristics may originate not only from changed levels of fibrinogen but also from altered fibrinogen properties, caused, e.g., by post-translational modifications (PTMs) [70,71,72,73,74,75]. A certain level of PTMs is necessary for the proper function of fibrinogen. Their overabundance, on the other hand, is known to alter the properties of fibrin clots, and some PTMs are connected with diseases [76]. It would be interesting, although challenging, to determine if episodes of the manifestation of fibrinogen mutations are connected with certain oxidative states of fibrinogen.

## 4. AαT331A SNP

AαT331A (rs6050) is the only known SNP in the αC-connector of fibrinogen [77,78]. This SNP is reported in 24.4–32.7% of the population worldwide [31,79,80] and is more common in the African (43.3%) and East Asian (43.2%) populations than in the South Asian (24.7%), European (23.6%), and American (21.8%) populations [79].

Fibrin clots formed by fibrin containing the AαA331 allele have more α-α cross-links than AαT331 fibrin (54% vs. 45% of cross-links), are stiffer, and have thicker fibrin fibers and fewer fibers per square unit than AαT331 fibrin. The SNP does not influence the level of fibrin fiber branching [81], or permeability of the clot [82]. More detailed analysis showed that the rate at which permeability decreases with increasing fibrinogen concentration is greater in individuals carrying AαA331 [82].

There are contradictions concerning the impact of AαT331A on the functional concentrations of fibrinogen in the blood. While Rasmussen-Torvik et al. [83] and Li et al. [84] did not record any change, Reiner et al. [85] reported decreased levels of functional fibrinogen among AαA331 carriers in European American but not in African American populations. AαT331A does not influence the level of immunological fibrinogen [85].

The AαA331 allele is associated with venous thromboembolism, pulmonary embolism, chronic thromboembolic pulmonary hypertension, atrial fibrillation, and spontaneous abortions at the beginning of pregnancy in women [84,86,87,88,89,90]. It is not associated with myocardial infarction, pulmonary thromboembolism, or stroke [84,87,91]. AαA331 is hypothesized to increase susceptibility to a thrombus embolization [87].

## 5. Summary

We reviewed mutations in the αC-connector of fibrinogen and cataloged as much clinically and structurally relevant information (location at the protein and nucleotide level, clinical manifestations, and laboratory characteristics) about each item as possible. We listed not only the subjects referred as patients but also all their available relatives. Our work revealed that the general COSMIC and dbSNP databases contain considerably more missense and synonymous mutations than the HFD, the database focused on clinically relevant mutations in fibrinogen. This, together with the information contained in the HFD, suggests that homozygous nonsense and frameshift mutations in the αC-connector are of clinical significance, unlike heterozygous mutations. We did not draw any conclusions about carriers of homozygous missense mutations as no such subjects were reported with regard to the αC-connector to the best of our knowledge.

Like other fibrinogen mutations, mutations in the αC-connector may manifest as bleeding or, less frequently, thrombotic phenotypes. Carriers of heterozygous mutations were often asymptomatic. Miscarriages and delayed wound healing were reported among αC-connector mutation carriers. No subject reported both bleeding and thrombotic phenotypes unless the thrombosis was induced by treatment. Interestingly, the thrombotic phenotype was observed only for mutations in the last position of the consensus sequence of the tandem repeat region.

## Figures and Tables

**Table 1:** **(previous page).** List of unique mutations in the αC-connector of fibrinogen and their positions in the protein (nascent chain, reference NP_068657.1) and coding (CDS of NM_021871.4) sequences. Champagne = complex fibrinogen Champagne au Mont d’Or. The table further reports CFD in relation to mutation zygosity: OK = no CFD; afib = afibrinogenemia; hypo = hypofibrinogenemia; dys = dysfibrinogenemia; hdys = hypodysfibrinogenemia; s = severe, mo = moderate; mi = mild; acq = acquired. Clinical manifestations: bl. = bleeding phenotype; thr. = thrombotic phenotype; mis. = miscarriage; DWH = delayed wound healing; asym. = asymptomatic; o = homozygous mutation; e = heterozygous mutation; c = compound mutation. T = clinical manifestation after treatment. See Table S2 for the extended version of this table, including information about each subject mentioned in the original literature, specification of their clinical manifestations, and genomic location of the mutation.

Position		Diagnosis			Clinical Manifestation					Ref
CDS	Protein	Homo	Hetero	Compound	bl.	thr.	mis.	DVH	asym.	
c.718C>T	p.Q240*	afib			o		o	o		[37]
c.743G>A	p.W248*	afib			o					[38,39]
c.786_789del4	p.E262Dfs*158	afib			o					[40,41]
c.811C>T	p.R271*		OK	afib: ? Bβp.S189T	c				e	[42]
c.835delA	p.T279Pfs*142		OK	afib: cis rs146387238	c				e	[43]
c.858_859insC	p.R287Efs*4	s hypo	OK		o		o			[44]
c.865C>A	p.P289T		dys		e				e	[45]
Champagne	Champagne			dys: ? c.510+36T>C		e				[32]
c.885G>A	p.W295*	afib; s hdys			o	o				[27,38,46]
c.888_894dup7	p.S299Lfs*57	afib			o					[41]
c.934delA	p.S312Afs*109	afib	OK; mi hypo	afib: cis RCV000017876.28	o				e	[38,47,48,49]
c.934_935insA	p.S312Kfs*42	afib								[50]
c.945delT	p.G316Efs*105	afib	OK		o				e	[28,38,47,48]
c.946G>T	p.G316*			afib: cis rs146387238						[38,51]
c.997A>T	p.S333C			dys: cis p.G32E	c					[52]
c.1001G>A	p.W334*	afib	OK	afib: cis p.K144Sfs*16; SNP	o, e	oT	o, e	o	e	[38,51,53]
c.1002G>A	p.W334*					o, oT			e	[54]
c.1025delG	p.G342Efs*79	afib		s hdys: trans p.T331A	o, e					[38,55]
c.1037delA	p.N346Tfs*75	afib			o					[38]
c.1039C>T	p.Q347*		OK	s hdys: cis rs146387238	c	cT	c			[56]
c.1040A>C	p. Q347P		OK; acq dys			e			e	[57]
c.1055delC	p.P352Lfs*69			afib: cis rs146387238						[38,51]
c.1119 G>A	p.W373*	mo hdys	OK		o				e	[58]
c.1199 C>T	p.S400F			dys: trans p.G36C	c				c	[59]

**Table 2:** Mutations in the αC-connector of fibrinogen assigned to the CFDs they cause. The positions within the nascent protein chain are used. Homozygous mutations are underlined. Champagne = complex fibrinogen Champagne au Mont d’Or; IVS4 = intronic mutation g.510+1G>T; 11 kb del = RCV000017876.28; s, acq = acquired, mo, and mi = severe, moderate, and mild hypofibrinogenemia and hypodysfibrinogenemia, respectively.

Afibrinogenemia	Hypofibrinogenemia	Dysfibrinogenemia	None
Q240*	R287Efs*4 (s)	P289T	R271*
W248*	S312Afs*109 (mi)	Champagne	T279Pfs*142
E262Dfs*158	[W334*;T331A];[T331A] (mi)	[G32E];[S333C]	R287Efs*4
[R271*(;)BβS189T]		Q347P (acq)	S312Afs*109
[T279Pfs*142];IVS4		[G36C;S400F];[T331A]	G316Efs*105
W295*		[G36C;S400F]	W334*
S299Lfs*57	**Hypodysfibrinogenemia**		[W334*;T331A];[T331A;BβR478K]
S312Afs*109	W295* (s)		Q347P
S312Kfs*42	[G342Efs*79;T331A] (s)		Q347*
[S312Afs*109];11kb del	[Q347*];IVS4 (s)		W373*
[G316*];IVS4	W373* (mo)		
G316Efs*105			
W334*			
[W334*;T331A]			
[W334*];[K144Sfs*16]			
G342Efs*79			
N346Tfs*75			
[P352Lfs*69];IVS4			

**Table 3:** Mutations in the αC-connector of fibrinogen assigned to their clinical manifestations. The positions within the nascent protein chain are used. Homozygous mutations are underlined. Champagne = complex fibrinogen Champagne au Mont d’Or; IVS4 = intronic mutation g.510+1G>T; DWH = delayed wound healing; T = state after treatment.

Bleeding	Thrombosis	Miscarriage	DWH	Asymptomatic
Q240*	Champagne	Q240*	Q240*	R271*
W248*	W295*	R287Efs*4	W334*	T279Pfs*142
E262Dfs*158	[W334*;T331A] (T)	W334*		P289T
[R271*(;)BβS189T]	W334* (T)	W334*		S312Afs*109
[T279Pfs*142];IVS4	Q347P	[Q347*];IVS4		G316Efs*105
R287Efs*4	[Q347*];IVS4 (T)			W334*
P289T	[G36C;S400F]			Q347P
S299Lfs*57	[G32E];[S333C]			Q347*
S312Afs*109				W373*
G316Efs*105				[G36C;S400F]
W334*				
[G342Efs*79;T331A]				
G342Efs*79				
N346Tfs*75				
[Q347*];IVS4				
W373*				
[G32E];[S333C]				
[G36C;S400F]				

**Table 4:** Polymerization characteristics of fibrinogen with mutations in the αC-connector of fibrinogen. Values designated by # were read out of the graph in the original report. * = fibrin still polymerizes; c.hetero = compound heterozygote; homo = homozygote; hetero = heterozygote; IVS4 = intronic mutation g.510+1G>T.

Position	Zygosity	Subject	Lag Phase [s]	V_max_ [U/s]	Final Turbidity [U]	Clottability	Ref.
p.R287Efs*4	homo	I:1	3x longer than control		0.075 at 150 min *		[44]
		#	1800				
		normal #	600	2.3 × 10^−5^	0.053		
p.[G32E];[S333C]	c.hetero	pac	prolonged		decreased		[52]
p.[G342Efs*79;T331A]	hetero	IV-1	240	0.08 × 10^−3^	0.1	21%	[55]
p.[G342Efs*79;T331A]	hetero	IV-3				23%	
None		III-2				95%	
		normal	180	1.15 × 10^−3^	0.7	96%	
p.[Q347*];IVS4	c.hetero	II.1	430	0.78 × 10^−4^	0.023		[56]
		normal	220	2.22 × 10^−4^	0.111		
p.Q347P	hetero	son	240	0.53 × 10^−4^	0.040	97%	[57]
		normal	150	1.04 × 10^−4^	0.064	98%	

## Data Availability

The data presented in this study are available in the article, Supplementary Materials, and original works.

## Supplementary Materials

The following are available online at https://www.mdpi.com/article/10.3390/ijms23010132/s1.
