# Supplementary material for: Extension of the Human Fibrinogen Database with Detailed Clinical Information—The αC-Connector Segment
Source: Int J Mol Sci. 2021 Dec 23;23(1):132. doi: 10.3390/ijms23010132 (PMC8745514; doi:10.3390/ijms23010132)
Supplement: Supplementary file 1 [file ijms-23-00132-s001.zip › SupplementaryFiguresText.pdf]

Figure S1. Complex compound mutation fibrinogen Champagne au Mont d'Or aligned with the wild-type fibrinogen on nucleotide (A; CDS of sequence NM_021871.4) and protein (B; nascent chain of sequence NP_068657.1). Numbering for the wild-type sequence, nascent chain is used for protein. Sequences were aligned with ClustalX.

A

```
Champagne    TGGACCTGGAAGTACTGGAAACCGAAACCCTGGGAGCTCTGGGACTGGAGGGACTGCAACCTGGAAACCTGGGAGCTCTGGACCTGGAAG
NM_021871.4  TGGAACTGGAAGTACTGGAAACCAAAACCCTGGGAGCCCTAG------------------------------------------------
               1020      1030      1040      1050

Champagne    TGCTGGAAGCTGGAACTCTGGGAGCTCTGGACCTGGAAGTACTGGAAACCGAAACCCTGGGAGCTCTGGGACTGGAGGGACTGCAACC
NM_021871.4  ------------------------------ACCTGGTAGTACCGGAA---------------------------------------CC
                                           1060      1070                                      1077
```

B

```
Champagne    SGPGSTGNRNPGSSGTGGTATWKPGSSGPGSAGSWNSGSSGPGSTGNRNPGSSGTGGTAT
NP_068657.1  SGTGSTGNQNPGSP--------RPGSTG-------------------------------T
               360       370                                           378
```

Figure S2. Mutation in the αC-connector of fibrinogen reported in the Human Fibrinogen Database, nascent chain numbering. Nonsense mutation are indicated by blue line, frameshift mutations by red line, missense mutations by magenta, mutations belonging to fibrinogen Champagne au Mont d'Or are green.

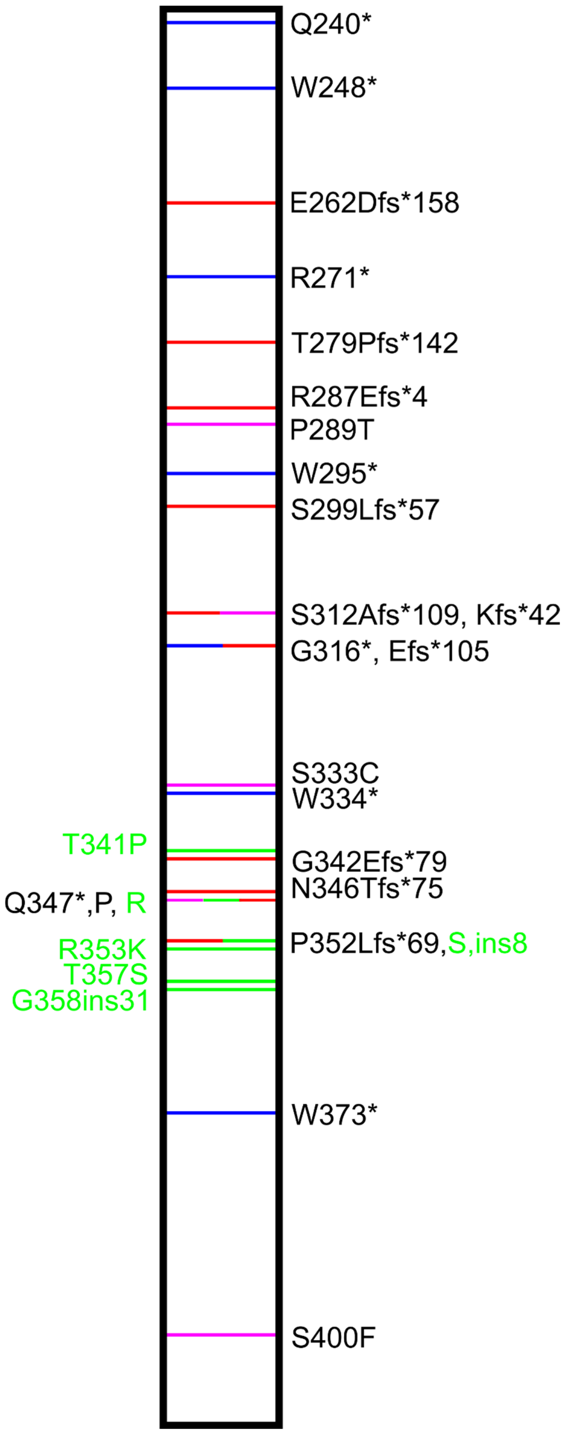

Supplementary Text 1: Explanation of adjustments in mutation positions reported in the HFD.

Fibrinogen **India**, patient BL-502 [1]: Mutation notation at the protein level was adjusted to follow the notation according to Den Dunnen et al [2]. The original designation for the nascent chain is p.E262_263fs*158.

Fibrinogen **Tunisia**, mutation in the Bβ chain [3]: Heterozygous substitution g.5118G>C resulting in the silent polymorphism S157S (mature chain) is reported. There is a lysine at position 157 of the nascent chain and a valine at position 157 of the mature chain. There is a guanine at position g.5118 (reference M64983 that is reportedly used in the work), in agreement with the original work. This corresponds to guanine 566 (CDS of NM_005141.5) that encodes serine 159 of the mature chain (i.e. serine 189 of the nascent chain); codon AGC. Thus, the reported mutation manifests as p.S189T (ACC) at the protein level.

Fibrinogen **France** [4]: The genomic sequence is referred to as c.DNA. The reported positions are c.[4110delA];[3200+1G>T]. The c.DNA of the *FGA* gene (reference NM_021871.4) has 2209 bp only. There is a guanine at position 3201 and an adenosine at position 4110 in the genomic sequence of the *FGA* gene (reference M64983).

Fibrinogen **Otago** [5]: Mutation notation at the nucleotide level was adjusted to follow the notation according to Den Dunnen et al [2]. The original designation is g.4133insC.

Fibrinogen **India**, patient BL-564 [1]: The mutation is reported as c.887_894dup7 and p.D277fs*59 (mature chain). Interval 887_894 contains eight nucleotides, not seven. Duplication of nucleotides c.887_894 (i.e., eight nucleotides) results in p.S280Tfs*125 (mature chain). Both c.887_893dup7 and c.888_894dup7 result in p.S280Lfs*57. We suggest that the latter possibility is correct as it is closer to the reported protein sequence. However, we are not entirely sure of the true nature of this mutation.

Fibrinogen **Switzerland**, patient B6 [6,7]: The work [7] reports the deletion of an adenosine at position g.4179 (reference M64984) that corresponds [6] to c.934 and is translated into p.S293Afs*109 (mature chain). The work [6] reports the identical mutation as in the work [8] where the genomic position of the deleted adenosine is reported as g.4209 (reference M64984, i.e., the same). At g.4179, which corresponds to c.904, there is a cytosine, and this nucleotide belongs to the codon of p.P302 (nascent chain). The original location does not agree with the reported position of the mutation at the c.DNA or protein level. Adenosine g.4209 corresponding to c.934 encodes p.S312 (nascent chain), and its deletion leads to p.S331Afs*109. Thus, the position on the genomic sequence was adjusted.

Fibrinogen **Podesin** [9]: The mutation is reported as g.6477del (exon 5) that encodes p.?300Afs*41. There is a guanine at position 6477 in the reference sequence M64984. (The authors do not specify the reference sequence used; thus, we assume that they used the most commonly used reference sequence.) The nucleotide 6477 belongs to exon 6 encoding the alternatively spliced chain Aα and thus does not encode the amino acid at position 300. Personal communication with the authors of the work [9], some of whom are authors of this paper, revealed that the sequence at https://pga.gs.washington.edu/data/fga/fga.ColorFasta.html was used as a reference. Position 6477

of the abovementioned sequence corresponds to position 4209 of the sequence M64984, and fibrinogen Podesin is identical with fibrinogen Iran [8] and Switzerland [6,7].

Fibrinogen **Turkey and Lebanon**, patients B5 and C4 [6,7]: The original work [7] reports a mutation at position g.4190delT (M64984) and an additional review [6] describes the mutation as c.945delT, resulting in p.G316Efs*104 (nascent chain). There is a cytosine at position 4190 of the M64984 sequence. Thymine is at position c.945 and its deletion encodes the mutation p.G316Efs*105 (nascent chain). Position c.945 corresponds to g.4220.

Fibrinogen **France**, patient A12 [6,10]: Formal correction of the genomic coordinates was done to follow the notation according to Den Dunnen et al. [2] Original designation: g.3121delAA.

Fibrinogen **United States** [6,10]: Adjustment was made to follow the 3'-rule as per Den Dunnen et al [2]. Original notation: g.4329delC, with cytosines at positions g.4328_4330.

Fibrinogen **Sumperk** [11]: The authors refer to mutations g.3447G>A encoding p.G13E (mature chain) and g.6540A>T encoding p.S314C. They use the genomic sequence M64982 as a reference.

There is a thymine at position g.3447 in M64982 (guanine is reported) that belongs to intron 4; therefore, it does not encode any amino acid. The authors do not use c.DNA as a reference because the commonly used sequence NM_021871.4 contains 2209 bp only. Glycine 13 is encoded by the codon GGA. Replacement of the first guanine by adenosine results in the codon AGA encoding an arginine. Replacement of the second guanine by adenosine results in the codon GAA encoding glutamic acid, the reported mutation. Thus, the reported mutations must be c.95G>A and g.1194G>A.

The second mutation is referred to as g.6540A>T. There is a guanine at position 6540 in the sequence M64982, while an adenosine is reported. Position 6540 is out of range of the c.DNA sequence NM_021871.4. There is a serine at position 314 (mature chain) encoded by AGC in the Aα chain of fibrinogen. Mutation of this adenosine to thymine results in cysteine (TGC), in agreement with the report. The positions of the mutations at the nucleotide level are then c.997A>T and g.4272A>T.

Fibrinogen **Madhia** [13]: There is no information on whether the mutation and SNP are on one allele or on both. Because both of them are reported in the father and two out of his four children, while neither the other two children nor the mother has any mutations, we treated the mutations as trans compound mutations.

Fibrinogen **Turkey** [12]: The chromosomal positions of the mutations are not mentioned. The work deals with a couple and their two sons. To be comprehensive, we designated one son as a patient and the other one as a son. Further, the zygosity of the mutations is specified; all of them are homozygous for the AαT331A SNP, the patient is homozygous for the AαW334* mutation, and the others are heterozygous for this mutation. See Figure for a graphical explanation of the deduction. Mother and son are heterozygous for the BβR478K SNP. Homozygous AαT331A indicates that all four paternal alleles within this family (two from each parent) contain this SNP. The BβR478K SNP is reported in both mother and son in the heterozygous state. The son inherited this SNP from the mother, and because all alleles contain the AαT331A SNP, these two SNPs will be on the same allele. The second allele of the mother and son must contain the mutation AαW334* and the SNP AαT331A. This agrees with the information that mother and son are heterozygous for AαW334* and BβR478K

and homozygous for AαT331A. It further indicates that the father must contain the allele p.[AαW334*;AαT331] because the son could not have inherited both alleles from the mother. The father's second allele contains AαT331A because he is homozygous for this mutation and no other mutations are reported for him. Finally, the patient contains two [AαW334*;AαT331A] alleles because he is homozygous for these mutations and each of his parents contains one such allele. There is no other way to satisfy the conditions reported in the original work, not accepting the hypothetical possibility that identical mutations would be formed in the second generation.

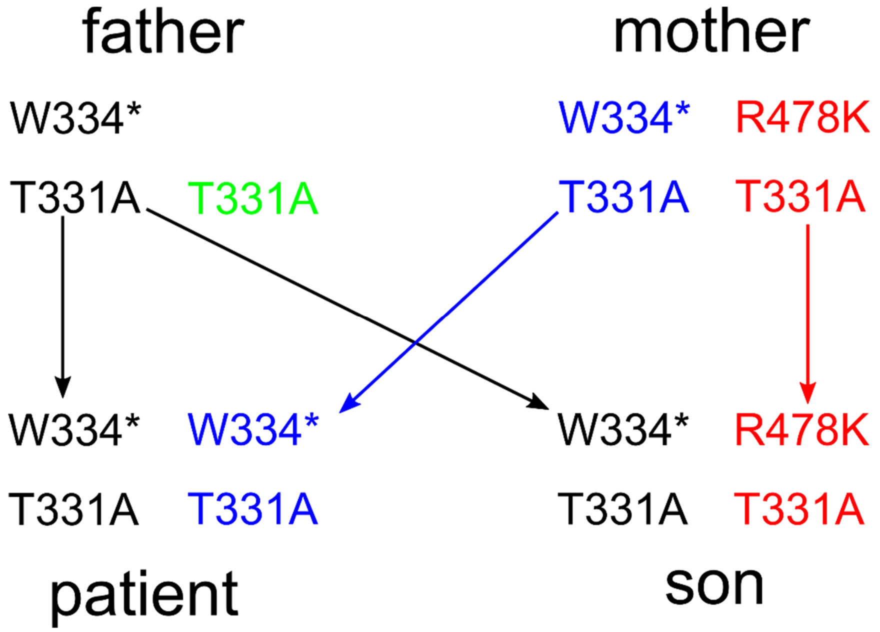

Fibrinogen **Austin** [14]: Data on the presence of the mutation p.S400F are absent for brother 4 and son 2. The work shows that this mutation is present in this family on the same allele as the mutation p.G36C. Because p.G36C is present in brother 4 and absent in son 2, we inferred that p.S400F is also present in brother 4 and absent in son 2.

Fibrinogen **India**, patient P16 [15,16]: The HFD lists the homozygous mutation p.A307V (nascent chain) in the Aα chain that is encoded by c.920C>T and reported by Mukaddam et al. in 2015. The original report deals with a mutation p.A307V in the Bβ chain. We did not find any other work by Mukaddam et al. describing a p.A307V mutation in the Aα chain of fibrinogen. Furthermore, this mutation is reported in the sixth exon, while the *FGA* gene has five exons in the major form. Exon 6 of the minor form of fibrinogen starts at position c.1892 and p.631 but the mutation is reported at position c.920, corresponding to p.307. This means that it does not belong to the minor form of the *FGA* gene. Both other fibrinogen genes, *FGB* and *FGG*, contain exon 6. A cytosine at position c.920 belonging to a codon encoding alanine at position p.307 (nascent chain) is found in the c.DNA of the *FGB* but not the *FGG* gene. The original work states that the mutation is known. A search in dbSNP revealed the mutation in the *FGB* gene (rs777451745) but not the *FGA* and *FGG* genes. Because of this, we excluded this mutation from any consideration in this review.

References

[1] Sumitha E, Jayandharan GR, Arora N, Abraham A, David S, Devi GS et al. Molecular basis of quantitative fibrinogen disorders in 27 patients from I ndia. Haemophilia 2013;19:611-8.

[2] Den Dunnen JT, Dalgleish R, Maglott DR, Hart RK, Greenblatt MS, McGowan-Jordan J et al. HGVS recommendations for the description of sequence variants: 2016 update. Hum Mutat 2016;37:564-9.

[3] Amri Y, Toumi NEH, Fredj SH, de Moerloose P. Congenital afibrinogenemia: Identification and characterization of two novel homozygous fibrinogen Aα and Bβ chain mutations in two Tunisian families. Thromb Res 2016;143:11-6.

[4] Angles-Cano E, Mathonnet F, Dreyfus M, Claeyssens S, de Mazancourt P. A case of afibrinogenemia associated with A-alpha chain gene compound heterozygosity (HUMFIBRA c.[4110delA] [3200 1G> T]). Blood Coagulation Fibrinol 2007;18:73-5.

[5] Ridgway HJ, Brennan SO, Faed JM, George PM. Fibrinogen Otago: a major α chain truncation associated with severe hypofibrinogenaemia and recurrent miscarriage. Br J Haematol 1997;98:632-9.

[6] Neerman-Arbez M, De Moerloose P. Mutations in the fibrinogen gene cluster accounting for congenital afibrinogenemia: an update and report of 10 novel mutations. Hum Mutat 2007;28:540-53.

[7] Neerman-Arbez M, De Moerloose P, Honsberger A, Parlier G, Arnuti B, Biron C et al. Molecular analysis of the fibrinogen gene cluster in 16 patients with congenital afibrinogenemia: novel truncating mutations in the FGA and FGG genes. Hum Genet 2001;108:237-40.

[8] Asselta R, Spena S, Duga S, Peyvandi F, Malcovati M, Mannucci PM et al. Analysis of Iranian patients allowed the identification of the first truncating mutation in the fibrinogen Bbeta-chain gene causing afibrinogenemia. Haematologica 2002;87:855-9.

[9] Štikarová J, Blatný J, Kotlín R, Suttnar J, Zapletal O, Pimková K et al. Novel homozygous fibrinogen Aα chain truncation causes severe afibrinogenemia with life threatening complications in a two-year-old boy. Thromb Res 2013;132:490-2.

[10] Neerman-Arbez M, De Moerloose P, Bridel C, Honsberger A, Schönbörner A, Rossier C et al. Mutations in the fibrinogen Aα gene account for the majority of cases of congenital afibrinogenemia. Blood, The Journal of the American Society of Hematology 2000;96:149-52.

[11] Kotlín R, Suttnar J, Cápová I, Hrachovinová I, Urbánková M, Dyr JE. Fibrinogen Šumperk II: dysfibrinogenemia in an individual with two coding mutations. Am J Hematol 2012;87:555-7.

[12] Simsek I, de Mazancourt P, Horellou M, Erdem H, Pay S, Dinc A et al. Afibrinogenemia resulting from homozygous nonsense mutation in A alpha chain gene associated with multiple thrombotic episodes. Blood Coagulation Fibrinol 2008;19:247-53.

[13] Amri Y, Jouini H, Becheur M, Dabboubi R, Mahjoub B, Messaoud T et al. Fibrinogen Mahdia: A congenitally abnormal fibrinogen characterized by defective fibrin polymerization. Haemophilia 2017;23:e340-7.

[14] Brennan SO, Laurie AD, Mo A, Grigg A. Novel fibrinogen mutations (Aα17Gly→ Cys and Aα381Ser→ Phe) occurring with a 312Thr→ Ala polymorphism: allelic phase assigned by direct mass measurement. Blood Coagulation Fibrinol 2015;26:882-6.

[15] Mukaddam A, Patil R, Jadli A, Chandrakala S, Ghosh K, Shetty S. Paradoxical bleeding and thrombosis in a patient with afibrinogenemia and fibrinogen Mumbai mutation. Am J Clin Pathol 2015;143:755-7.

[16] Mukaddam A, Kulkarni B, Jadli A, Ghosh K, Shetty S. Spectrum of mutations in Indian patients with fibrinogen disorders and its application in genetic diagnosis of the affected families. Haemophilia 2015;21:e519-23.
